# BUB1B (BUB1 Mitotic Checkpoint Serine/Threonine Kinase B) promotes lung adenocarcinoma by interacting with Zinc Finger Protein ZNF143 and regulating glycolysis

**DOI:** 10.1080/21655979.2021.2013108

**Published:** 2022-01-22

**Authors:** Xiaolei Zhou, Yanli Yuan, Hongping Kuang, Bingxiang Tang, Hui Zhang, Manlin Zhang

**Affiliations:** a Department of Respiratory and Critical Medicine, Henan Chest Hospital, Zhengzhou, P.R. China; b Department of Respiratory and Critical Medicine, The First Affiliated Hospital of Zhengzhou University, Zhengzhou, P.R. China

**Keywords:** BUB1B, ZNF143, lung adenocarcinoma, transcriptional activator, glycolysis

## Abstract

Lung adenocarcinoma (LUAD) is one of the most common causes of cancer death in men. BUB1B (BUB1 mitotic checkpoint serine/threonine kinase B) has been reported to contribute to the initiation and development of several cancers. Here, we aimed to explore the potential role of BUB1B in LUAD. We found BUB1B was upregulated in LUAD, suggesting its potential role as a biomarker for LUAD diagnosis. Significantly, LUAD patients with high BUB1B expression had a shorter survival time than those with low BUB1B expression. Knocking-out BUB1B resulted in suppression of cell proliferation, migration, and invasion *in vitro*, and inhibition of tumor growth in the xenograft experiment. Further analysis revealed that BUB1B regulates glycolysis in LUAD and interacting with ZNF143 in LUAD cells. The interaction was demonstrated by silencing ZNF143, which led to a decrease in proliferation, migration, and invasion in LUAD cells, whereas overexpressing BUB1B had the opposite effects. Our study suggested that the ZNF143/BUB1B axis plays a pivotal role in LUAD progression, which might be a potential target for LUAD management.

## Graphical abstract

**Figure:**
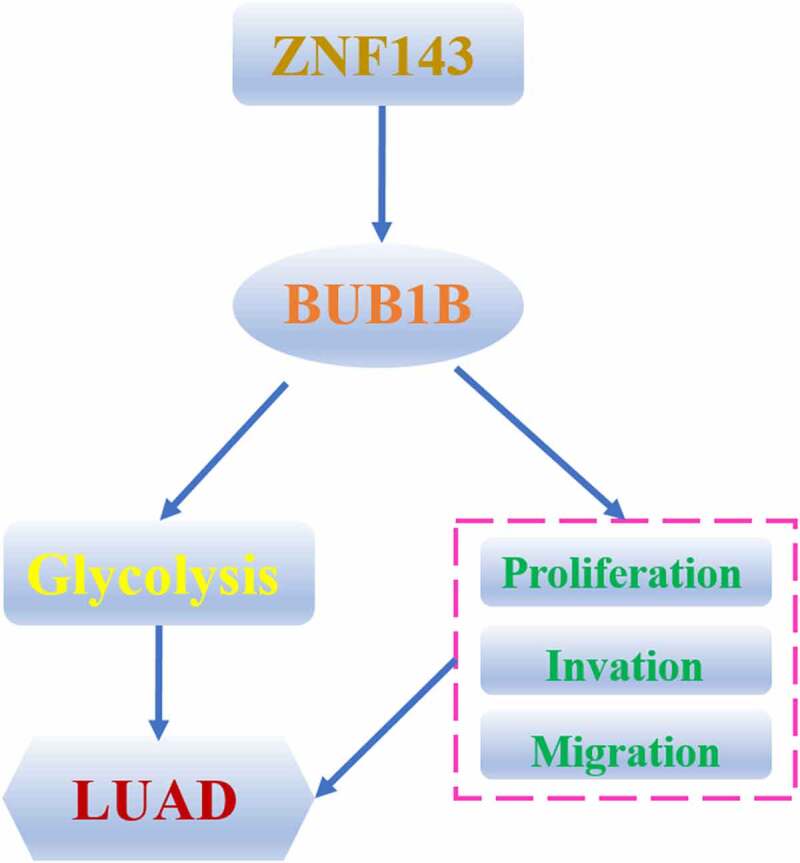

## Introduction

Lung cancer is the biggest contributor in cancer-associated male death (17.6%) [1]. Accounting for roughly 40% of lung cancer patients, lung adenocarcinoma (LUAD) represents a major cause of cancer mortality worldwide [2]. Despite significant advancements in the diagnosis and treatment of LUAD in the last decade [3], the overall survival (OS) and disease-free survival (DFS) rates of LUAD patients are kept at a stagnant low level, primarily owing to the heterogeneity nature of LUAD and the fact that LUAD is often diagnosed at an advanced stage [4]. In-depth investigation on the pathogenesis and tumorigenesis of LUAD is a prerequisite to develop novel diagnostic and therapeutic biomarkers for a better patient outcome.

Previous reports had suggested that chromosomal instability is often associated with human cancers [5–7]. To prevent errors during mitotic, checkpoints exist in the cell cycle to ensure the proper attachment of chromosomes to the spindle prior to the initiation of the anaphase [8] [9]. Mitotic checkpoint serine/threonine-protein kinase BUB1 (BUB1B), a member of the BUB1 proteins, regulates the checkpoint by phosphorylating the mitotic checkpoint complex members and activating the spindle checkpoint [10]. An aberrant expression of BUB1B had been reported in various cancers including ductal breast carcinoma (DBC) [11], glioblastoma (GBM) [12], and pancreatic ductal adenocarcinoma (PDAC) [13]. Functional consequences of aberrant expression of BUB1B had also been demonstrated. For example, Qiu *et al*. revealed that BUB1B overexpression facilitates the malignancy of hepatocellular carcinoma via activation of the mTORC1 axis [14]. Upregulation of BUB1B also promoted the proliferation of prostate cancer through the regulation of MELK transcription [15]. However, the potential mechanism of BUB1B dysregulation in LUAD remains poorly understood.

Transcription factors (TFs) are proteins that regulate tumor progression by promoting or inhibiting downstream genes [16] [17]. Dysregulation of multiple TFs had been associated with LUAD development and metastasis [18]. ZNF143 (Zinc Finger Protein 143) [18] is a transcriptional activator that dynamically binds to a subset of its interphase sites during mitosis. Moreover, ZNF143 is also a potential binding partner of BUB1B according to the TRRUST database. However, the role of the ZNF143/BUB1B axis is not fully understood in the context of LUAD carcinogenesis.

We investigated the underlying mechanism of BUB1B dysregulation and the role of the ZNF143/BUB1B axis in LUAD. We discovered that BUB1B acts as an activator in glycogen metabolism via being regulated by ZNF143. Moreover, we verified that BUB1B is remarkably upregulated in LUAD cell lines and tissues. We also characterized the clinical relevance and biological implications of BUB1B and revealed a role of the ZNF143-BUB1B axis in the progression of LUAD. Thus, BUB1B can be used as a novel diagnosis and therapeutic biomarker for LUAD.

## Methods and materials

### Clinical LUAD samples collection

Pairs of fresh frozen LUAD and paracancerous normal tissues (n = 90) were collected at Henan Chest Hospital. The detailed clinicopathological records were collected (Tab.S1) and all patients signed the informed consent before initiating this study. After surgery, patients were monitored regularly via telephone interviews. The study protocol was approved by the Ethics Committee of Henan Chest Hospital (HCH2019035).

### Cell culture and transfection

Human LUAD cell lines including H460, SK-LU-1, NCI-H1299, A549, and SK-LU-1 and 16HBE (normal bronchial epithelial cell line) were obtained from the Chinese Academy of Sciences (Shanghai, China). All cell lines routinely maintained in RPMI1640 (Gibco, Carlsbad, CA, USA) or DMEM medium (Gibco, Carlsbad, CA, USA) supplemented plus 10% (v/v) fetal bovine serum (FBS, Gibco, Carlsbad, CA, USA) and 1% penicillin and streptomycin in the incubator at 37°C with 5% CO_2_. The si-RNAs encoding BUB1B and ZNF143 and pCD-BUB1B vector were obtained from Gene Pharma (Shanghai, China). The si-RNAs were transfected into A549 and SK-LU-1 cells using Lipofectamine 2000 (Thermo Fisher Scientific, Waltham, MA, USA), respectively. Besides, sh-BUB1B (Gene Pharma, Shanghai, China) for stable knockdown of BUB1B were applied for the in vivo injection.

### TCGA and GEO datasets analysis

The LUAD mRNA expression profiles and corresponding clinicopathological records were downloaded from the TCGA database. To acquire the BUB1B-dependent expression profile, three GEO datasets, GSE7670, GSE10072, and GSE19188, were used. OS and DFS analysis were performed by Kaplan-Meier survival analysis and log-rank tests.

### Quantitative PCR (qRT-PCR)

Total RNA was isolated from LUAD cultured cell lines and tissues, using Trizol solution (Takara, Kusatsu, Japan), then reverse transcribed with Prime-Script RT reagent Kit (Takara, Kusatsu, Japan). The cDNAs (Genepharma, Shanghai, China)were then used for real-time PCR (RT-qPCR) on a 7500 Fast Real-Time PCR System (Applied Biosystems, Foster City, CA, USA) using SYBR Green Realtime PCR Master (Toyobo, China) under universal cycling conditions. GAPDH served as an internal control. Relative mRNA expression was normalized to actin levels using the 2^−ΔΔCt^ quantification method. The sequences of primers used in this study are listed below:

BUB1B: 5′-GCGGCGGTGAAGAAGGAAGGG-3′(Forward),

5′-TGCCAGTGCTCCCTGAAGCG-3′(Reverse);

GAPDH: 5′-ACCCAGAAGACTGTGGATGG-3′ (Forward),

5′-TTCAGCTCAGGGATGACCTT-3′ (Reverse);

### Western blot

Relative protein were extracted from tissue samples or cultured cells, and protein concentration was quantified by Lowry’s protein assay (Bio-Rad, Hercules, CA, USA) [19]. All protein samples were separated by 10% SDS-PAGE and then transferred onto polyvinylidene fluoride (PVDF) membranes.The primary antibodies used in this study were anti-BUB1B(#4116, Cell Signaling Technology, Beverly, MA, USA), anti-ZNF143(#PA5-72,658, Invitrogen, Waltham, MA, USA), anti-GLUT1(#ab115730, Abcam, Cambridge, UK), anti-LDHA(#ab101562, Abcam, Cambridge, UK), anti-PKM2 (#ab137852, Abcam, Cambridge, UK), anti-HK2 (#ab137852, Abcam, Cambridge, UK) and anti-Actin (#23,600-1-AP, Proteintech, China) antibodies. Western blot analysis was analyzed by Image J software.

### Cell Counting Kit-8 assay

Cellular proliferative potential was assessed by cell counting CCK-8 assay (Biosharp, Hefei, China) [19]. Concretely, the transfected A549 and SK-LU-1 cell lines were seeded into 96-well plates with 1000–2000 cells/well and conventionally cultured for 5 days. Then, each well added 10 μl of CCK-8 reagent for 4 hours at 37°C before measuring absorbance at 450 nm of each well.

### Colony formation assay

The transfected cell lines were plated into a 6-well plate and fostered for 14 days. Cells were fixed with 75% ethanol and stained with a crystal violet solution (Sigma, USA). The colony in which cell clusters were more than 50 cells was counted under a light microscope [19].

### 5-Ethynyl-2ʹ-deoxyuridine (EdU) staining assay

The transfected cell lines were plated into a 24-well plate and cultured for 1 day. Subsequently, DNA synthesis activity in LUAD cells was performed with an EdU assay kit (Ribobio, Guangzhou, China). EdU-positive cells were counted in three random fields for each well.

### Transwell assay

Cell invasive capability was evaluated by the transwell chambers(Corning, Corning, NY, USA) [19]. The insert used Matrigel-coated membranes (BD Biosciences, San Jose, CA, USA) in 24-well invasion chambers. We planted the cells into the upper chamber and cultured them with 200 μl DMEM with no addition of FBS; however, we filled the lower chamber with 500 μl complete medium containing 10% FBS, which attracted the cells across the Transwell membrane into the lower chamber. After incubation for 36 h, the invasive cells were fixed and stained with 0.5% crystal violet and then counted.

### Wound-healing assay

The transfected A549 and SK-LU-1 cell lines were plated into 6-well plates. After culturing for 1 day in serum-free media, the cells almost reached 90% confluence.Then the artificial wound was scraped using a 200 μl pipette tip in the center of the well. The streaked cells were washed with PBS to remove non-adherent cells. After culturing for 48 h, the distance between the wound sides was measured [19].

### Tissue microarray (TMA) construction and Immunohistochemistry (IHC)

All paraffin-embedded LUAD tissues collected from 90 suffers were used for TMA construction and IHC [20]. The TMA sections which IHC staining was performed on were incubated with anti-BUB1B(#4116, Cell Signaling Technology, Beverly, MA, USA) at 1:100. The final staining score was determined by the intensity (range 0–3+: 0, negative; 1+, weak; 2+, intermediate; and 3+, strong) and the percentage of staining cells(range 1+–4+: 1+, < 25%; 2+, 25%–50%; 3+, 50%–75%; and 4+, > 75%). A final score < 4 was reckoned as low expression and the other scores were taken as high expression.

### In vivo *xenograft model*

All animal experiments were in agreement with the institutional guidelines of Henan Chest Hospital. 4-week-old Male BALB/c nude mice were purchased from the Chinese Academy of Science (Shanghai, China) [19]. For the xenograft model study in vivo, 5 × 10^6^ of A549 cells transfected with sh-BUB1B or sh-NC were subcutaneously implanted into nude mice(n = 6 per group). Tumor growth was recorded once a week with caliper measurements (tumor volume = width^2^ × length×1/2) and a bioluminescence imaging system (Xenogen Corp., Alameda, CA, USA). Five weeks later, each mice tumor was obtained and weighed, followed by IHC staining for Ki-67 (Cell signaling technology, Beverly, MA, USA).

### Luciferase reporter assays

WT or mutated BUB1B-promoter was cloned into the pGL3-Luc reporter vector (Genepharma, Shanghai). LUAD cell lines were plated into 24-well plates and transfected with ZNF143 plasmids using Lipofectamine 2000 (Invitrogen, Waltham, MA, USA) after 24 h. After 48 h, luciferase activity was measured by using the Dual-Luciferase reporter assay system(Genepharma, Shanghai) as the manufacturer’s instructions [20].

### Chromatin immunoprecipitation(Chip) assays

The A549 and SK-LU-1 cells stably expressing 3xFlag-tagged ZNF143 had been cloned. Cells(2 × 10^6^) were preincubated with a dimethyl 3,3ʹ-dithiobispropionimidate-HCl solution (5 mM; Sigma, USA) for 30 min on ice and then treated with 1% formaldehyde at room temperature for 15 min. ChIP-enriched DNA samples were visualized by PCR and electrophoresed on a 2% agarose gel stained with ethidium bromide. Input DNA (1%) used for ChIP with individual antibody served as the control.

### Lactate production and glucose uptake

The A549 and SK-LU-1 cells were planted in six-well pates for measurement after 24 h starvation. Then the supernatants of these cells were collected for further examination. The glucose level and the lactate level was quantified by glucose assay kit (Sigma-Aldrich, USA) and the Lactate Assay kit (BioVision, USA) following the manufacturer’s protocols. Each experiment was performed in triplicates and repeated three times [20].

### Statistical analyses

All statistical results were carried out with SPSS 22.0 or Graphpad 8.4.0 software. Data in this study were represented as mean ± standard deviation (SD). All experiments were repeated independently at least three times. Unpaired two-tailed student’s t or Chi-square test was used to analyze the statistical differences between the two groups. Pearson’s correlation coefficient was employed to test the correlation between BUB1B and ZNF143 mRNA expression. *p < 0.05, **p < 0.01, ***p < 0.001.

## Results

LUAD is one of the most common causes of cancer death in men. BUB1B has been reported to contribute to the initiation and development of several cancers.In this paper, We found that BUB1B is up-regulated in LUAD and a biomarker for prognosis outcome by bioinformatic analysis. The same results appeared also in LUAD patients. BUB1B deletion suppressed LUAD malignancy in vitro and inhibited tumor growth in vivo through regulating glycolysis. Furthermore, the results indicated that ZNF143 modulates BUB1B in LUAD cells and ZNF143 enhances the tumorigenicity of LUAD cells in vivo by controlling BUB1B. High ZNF143 expression indicated poor prognosis like BUB1B in LUAD. Our study suggested that the ZNF143/BUB1B axis plays a pivotal role in LUAD progression, which might be a potential target for LUAD management.

### Upregulation of BUB1B in LUAD and its potential as a biomarker for prognosis outcome by bioinformatic analysis

**Figure 1.**
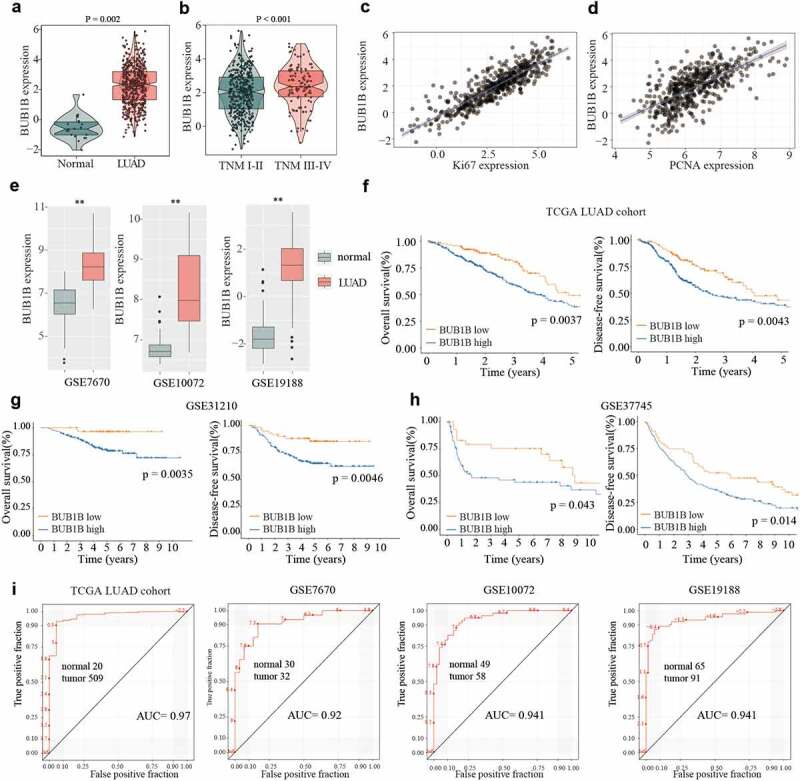
BUB1B is upregulated in LUAD, linked to the dismal outcome and a recommended biomarker for LUAD diagnosis by bioinformatic analysis. (a) The mRNA expression of BUB1B in normal tissues and LUAD tissues from the TCGA database. (b) The BUB1B expression in different TNM stages in the TCGA LUAD cohort. (c, d) The correlation between BUB1B expression and Ki67 (c) or PCNA (d) expression in the TCGA LUAD cohort. (e) The expression of BUB1B in normal tissues and LUAD tissues from the GSE7670, GSE10072, and GSE19188. (F, G, H) The overall survival and disease-free survival curves of LUAD patients with low and high BUB1B expression from the TCGA database(f), GSE31210(g), and GSE37745(h). (i) The prognostic utility of BUB1B expression from the TCGA LUAD cohort, GSE7670, GSE10072, and GSE19188. **p < 0.01.

To determine the expression of *BUB1B* in LUAD, we first searched the TCGA database. We found that *BUB1B* exhibited higher expression levels in LUAD tissues compared with adjacent normal tissues (Figure 1a). Furthermore, the *BUB1B* expression was drastically enhanced in tumors of advanced TNM stages (Figure 1b). Notably, a significant positive correlation was found between the *BUB1B* expression and the Ki-67 level (Figure 1c) or PCNA expression (Figure 1d). Moreover, a higher *BUB1B* expression in LUAD tissues was also found by examining the GEO database (Figure 1e). Next, we aimed to correlate the *BUB1B* expression level with the OS and DFS rates in LUAD patients. We found that higher *BUB1B* expression was associated with worse overall survival and recurrence-free rate in the TCGA LUAD cohort (figure 1f), GSE31210 (Figure 1g), and GSE37745 (Figure 1h). This suggested a diagnostic value of *BUB1B* expression. To that end, we plotted the receiver-operating characteristic (ROC) curve and found that BUB1B is a qualifiable as a biomarker for LUAD diagnosis with an AUC of 0.970 in the TCGA LUAD cohort, 0.920 in the GSE7670, 0.941 in the GSE10072, and 0.941 in the GSE19188, respectively. Collectively, these data suggest that *BUB1B* is significantly overexpressed in LUAD, which can be used as a biomarker for LUAD diagnosis.

### BUB1B is significantly up-regulated in LUAD tissues and predicts poor outcome in LUAD patients

**Figure 2.**
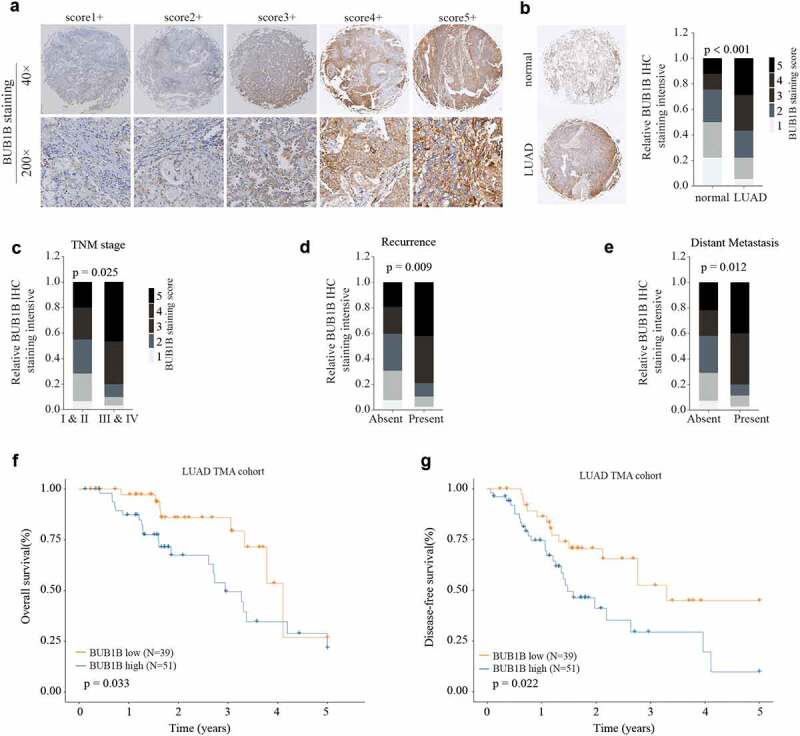
BUB1B is significantly increased in LUAD tissue and predicts poor outcome in LUAD patients. (a) Representative BUB1B IHC staining with different BUB1B staining scores in LUAD. (b) Representative BUB1B IHC staining in LUAD tissues and adjacent non-tumor normal tissues (left) and comparison of IHC staining score distributions between them (right). (c, d, e)Representative BUB1B IHC staining in LUAD tissues with different TNM stages(c), absence or presence of recurrence(d), and absence or presence of distant metastasis(e). (f, g) Kaplan-Meier analysis of OS(f) and DFS (g) in LUAD patients with low or high BUB1B expression.

To validate the bioinformatic analysis, we collected both LUAD tissues and paracancerous tissues to examine the protein abundance of BUB1B by IHC staining. Our data showed that protein level of BUB1B was drastically higher in LUAD tissues compared to normal tissues (Figure 2a,b). In line with the mRNA analysis, the BUB1B protein abundance is correlated with the tumor stage (Figure 2c), recurrence rate (Figure 2d), and distant metastasis (Figure 2e) in LUAD patients. Specifically, LUAD patients with high BUB1B levels had shorter OS and DFS than those with low BUB1B levels (figure 2f,g). Thus, these data validated the results of mRNA analysis and further indicated the potential of BUB1B for LUAD diagnosis.

### BUB1B deletion suppresses LUAD malignancy in vitro and inhibits tumor growth in vivo

**Figure 3.**
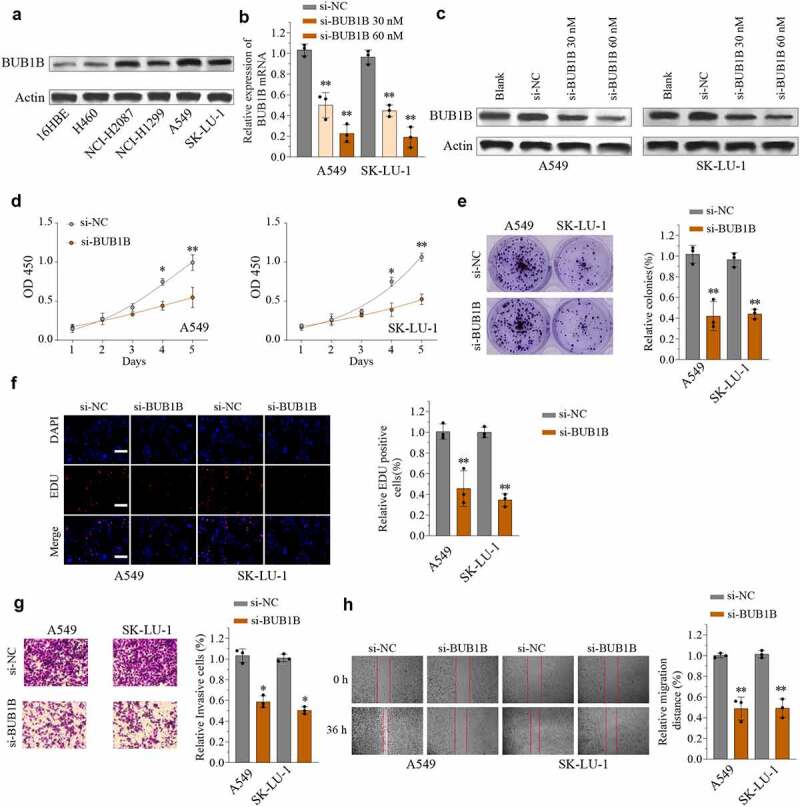
BUB1B deletion suppresses LUAD tumorigenicity in vitro. (a) Western blot analysis for BUB1B expression in LUAD cell lines including H460, SK-LU-1, NCI-H1299, A549, and NCI-H2087 and 16HBE. (b, c) qRT-PCR (b) and Western blot analysis (c) for BUB1B expression in the A549 or SK-LU-1 cells which were transfected with si-NC, 30 Mm siRNA targeting BUB1B(si-BUB1B), or 60 mM si-BUB1B. (d) CCK-8 proliferation assays in BUB1B-knockdown A549 (left) or SK-LU-1 (right) cells. (e) Colony formation assays in BUB1B-knockdown A549 or SK-LU-1 cells. (f) Representative immunofluorescence photos of EDU positive cells in BUB1B-knockdown A549 or SK-LU-1 cells. (g) Cell invasion analysis in BUB1B-knockdown A549 or SK-LU-1 cells. (i) Wound-healing assays in BUB1B-knockdown A549 or SK-LU-1 cells. *p < 0.05, **p < 0.01.

We next used a cellular model to study the functional role of BUB1B. First, the protein abundance of BUB1B was higher in LUAD cell lines compared to normal human bronchial epithelial 16HBE cells (Figure 3a). Among the LUAD cell lines, A549 and SK-LU-1 displayed strong BUB1B expression and were further used to generate stably transfected siRNA-BUB1B clones. Western blot revealed that si-BUB1B effectively reduced BUB1B expression in both A549 and SK-LU-1 cells in a dose-dependent manner (Figure 3b,c). Next, we assessed the impact of reducing BUB1B expression on cell proliferation migration and invasion. As expected, knocking-down BUB1B resulted in a significant inhibition of LUAD cell proliferation in both cell types, as demonstrated by CCK-8 cell proliferation assay (Figure 3d), colony formation (Figure 3e), and EDU/DAPI immunofluorescence staining (figure 3f). Likewise, depletion of *BUB1B* diminished the ability of A549 and SK-LU-1 cells to invade (Figure 3g) and migrate (Figure 3h). On the contrary, Overexpressing BUB1B promotes the proliferation(Fig.S3A), invasion(Fig.S3B), and migration (Fig.S3C) of A549 and SK-LU-1 cells in LUAD.

**Figure 4.**
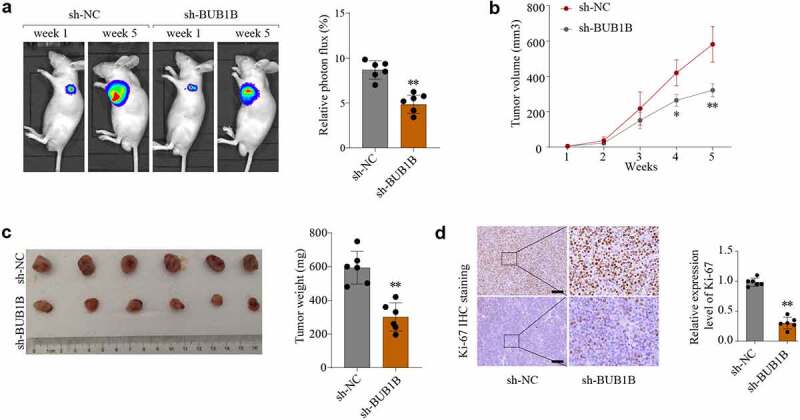
BUB1B knockdown inhibits lung tumor growth in vivo. (a) Representative bioluminescence imaging of nude mice and lung tumor tissues from control or sh-BUB1B group at week 1 and week 5. (b) Growth curves of tumor from control or sh-BUB1B group every week. (c) Tumor weight from control or sh-BUB1B group at week 5. (d) Ki-67 expression of tumor sections from the control or sh-BUB1B group. scale bar = 200 μm. *p < 0.05, **p < 0.01.

To investigate the contribution of *BUB1B* to tumor growth *in vivo*, a xenograft tumor model was established and sh-NC and sh-BUB1B was intratumorally injected. As shown in Figure 4a-C, the average tumor volume and weight was lower in the BUB1B-knockdown group compared to that in the control group. These findings were further confirmed by Ki-67 IHC staining (Figure 4d) and BUB1B IHC staining expression analysis (Fig.S4A). These observations suggested that BUB1*B* is an oncogene in LUAD and that targeting BUB1B may effectively delay LUAD progression.

### BUB1B regulates glycolysis in LUAD

**Figure 5.**
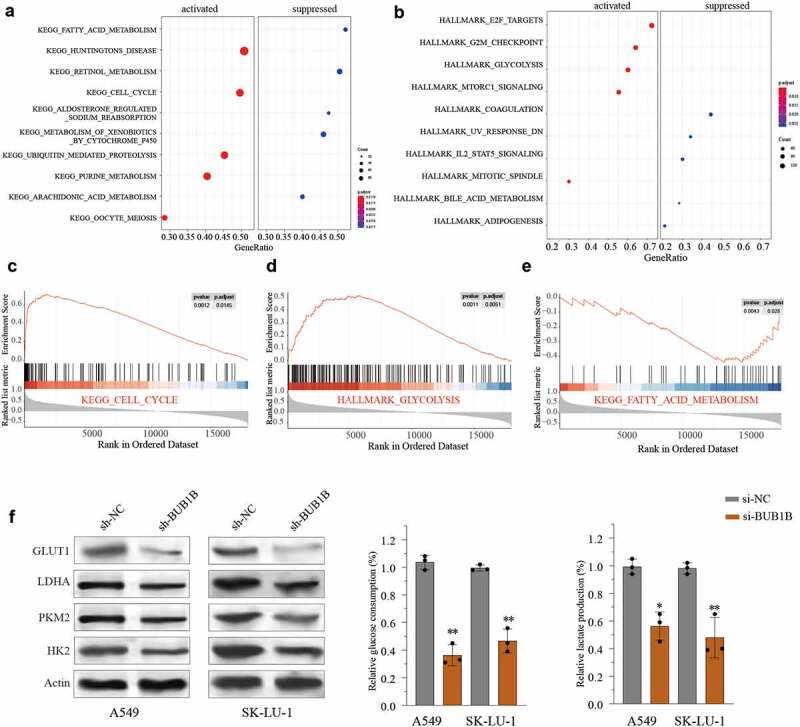
BUB1B regulates glycolysis in LUAD.(a-b) KEGG (a) and HALLMARK (b) analysis of the top 2000 genes with BUB1B overexpression in the LUAD TCGA cohort. (c–e) GSEA of the relationship between BUB1B expression and cell cycle (c), glycolysis (d), and fatty acid metabolism (e). (f)Western blot analysis for GLUT1, LDHA, PKM2, and HK2 expression in BUB1B-knockdown A549 or SK-LU-1 cells. (g, h)The glucose consumption (g) and the lactate production (E) in BUB1B-knockdown A549 or SK-LU-1 cells. **p < 0.01.

To further understand the potential function of *BUB1B* in LUAD, we performed KEGG and HALLMARK analysis using the top 2000 genes whose expressions are highly correlated with that of BUB1B from the TCGA LUAD cohort. We found that a high BUB1B expression was associated with remarkable activation of the cell cycle (Figure 5a) and glycolysis pathway (Figure 5b), and suppression of the fatty acid metabolism pathway (Figure 5a). Likewise, Gene Set Enrichment Analysis (GSEA) indicated that *BUB1B* expression was positively correlated with cell cycle and glycolysis pathway (Figure 5c,d) and inversely correlated with fatty acid metabolism pathway (Figure 5e). Thus, we next tested the impact of high *BUB1B* expression on glycolysis by transfecting A549 or SK-LU-1 cells with sh-BUB1B and sh-NC. qPCR (Fig.S1A, B) and Western blot (figure 5f) showed that silencing BUB1B noticeably decreased the expression of GLUT1, LDHA, PKM2, and HK2 in LUAD cell lines. Consistent with the expression data, a lower level of BUB1B also resulted in a reduced glucose uptake (Figure 5g) and lactate production (Figure 5h) in LUAD. Hence, it is likely that *BUB1B* plays a carcinogenic role by activating glycolysis in LUAD.

### ZNF143 modulates BUB1B in LUAD cells

**Figure 6.**
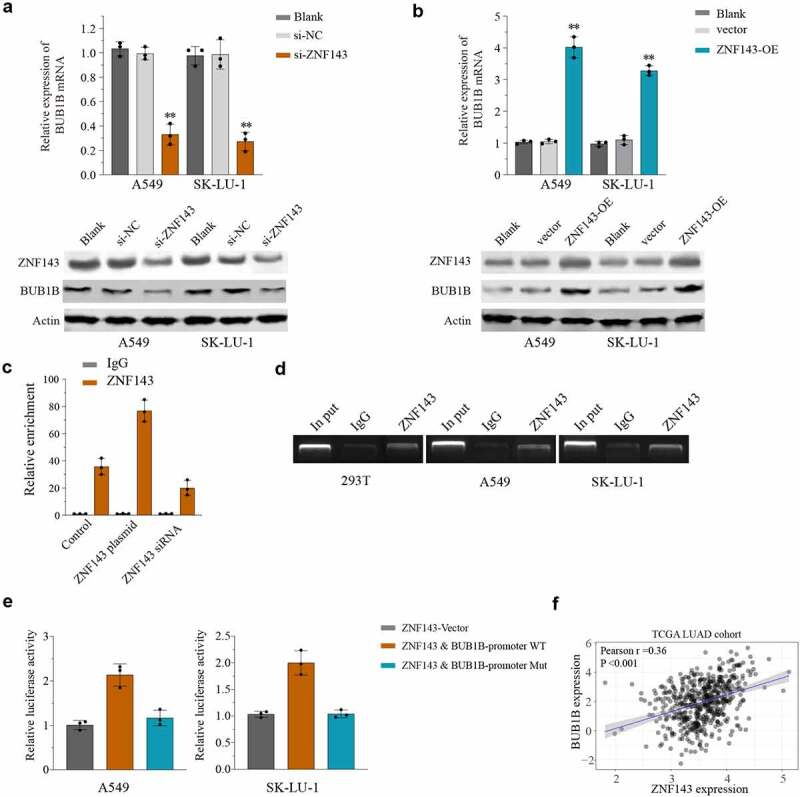
BUB1B interacts with ZNF143 in LUAD cells. (a, b) qRT-PCR analysis for BUB1B expression in ZNF143-knockdown (a) and ZNF143-overexpressing (b)A549 or SK-LU-1 cells. (c) CHIP assay of BUB1B with ZNF143 plasmid or siRNA in 293 T cells. (d) RNA pull-down assay in 293 T, A549 or SK-LU-1 cells. (e) Luciferase reporter assay of ZNF143 transcriptional activity in A549 or SK-LU-1 cells. (f) The correlation between BUB1B expression and ZNF143 expression in the TCGA LUAD cohort. **p < 0.01.

To further determine the mechanism by which BUB1B functions in LUAD, we performed a search using the TRRUST database and found that ZNF143 is a putative binding partner of BUB1B. The potential association was then studied by qPCR and Western blot, which showed that silencing ZNF143 resulted in a significant decrease of the BUB1B, mRNA(Figure 6a) and protein(Figure 6b) level in LUAD cells, whereas overexpressing BUB1B had the opposite effects. Moreover, an *in vivo* co-expression pattern was also confirmed by CHIP assay in 293 T cells (Figure 6c). Importantly, ZNF143 was capable of pulling down BUB1B in 293 T, A549, and SK-LU-1 cells (Figure 6d). In addition, the luciferase reporter assay demonstrated that ZNF143 enhanced the relative luciferase activity in LUAD cells transfected with WT BUB1B-promoter, but not the mutated BUB1B-promoter (Figure 6e). We noticed a significant positive correlation between BUB1B and ZNF143 in LUAD through Pearson’s correlation analysis (figure 6f). Together these results proved that ZNF143 modulates BUB1B in LUAD cells.

### ZNF143 enhances the tumorigenicity of LUAD cells in vivo by controlling BUB1B

**Figure 7.**
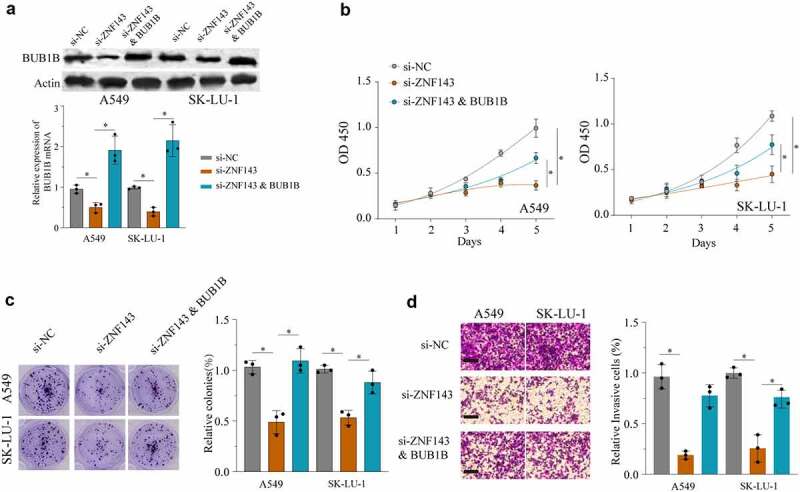
ZNF143 enhances the tumorigenicity of LUAD cells by controlling BUB1B. (a) Western blot analysis for BUB1B expression in ZNF143-knockdown A549 or SK-LU-1 cells co-transfected with BUB1B. (b) CCK-8 proliferation assays in ZNF143-knockdown A549(left) or SK-LU-1 (right) cells co-transfected with BUB1B. (c) Colony formation assays in ZNF143-knockdown A549 or SK-LU-1 cells co-transfected with BUB1B. (d) Cell invasion analysis in ZNF143-knockdown A549 or SK-LU-1 cells co-transfected with BUB1B. scale bar = 50 μm.*p < 0.05, **p < 0.01.

Ablation of ZNF143 using si-ZNF143 led to a significant inhibition of *BUB1B* expression in A549 cells and SK-LU-1 cells, while co-transfection with both si-ZNF143 and a BUB1B-overexpressing vector mildly rescued the BUB1B expression (Figure 7a). Next, we sought to determine whether ZNF143 promotes tumorigenesis by regulating BUB1B in LUAD. As evidenced in Figure 7b-D, knocking-down ZNF143 remarkedly inhibited the proliferation, migration, invasion, glucose uptake (Fig.S2A) and lactate production (Fig.S2B) of A549 and SK-LU-1 cells. However, these effects were counteracted by BUB1B overexpression. Taken together, these data demonstrated that ZNF143 exerts the carcinogenic role, at least partially through modulating BUB1B in LUAD.

### High ZNF143 expression indicates poor prognosis like BUB1B in LUAD

**Figure 8.**
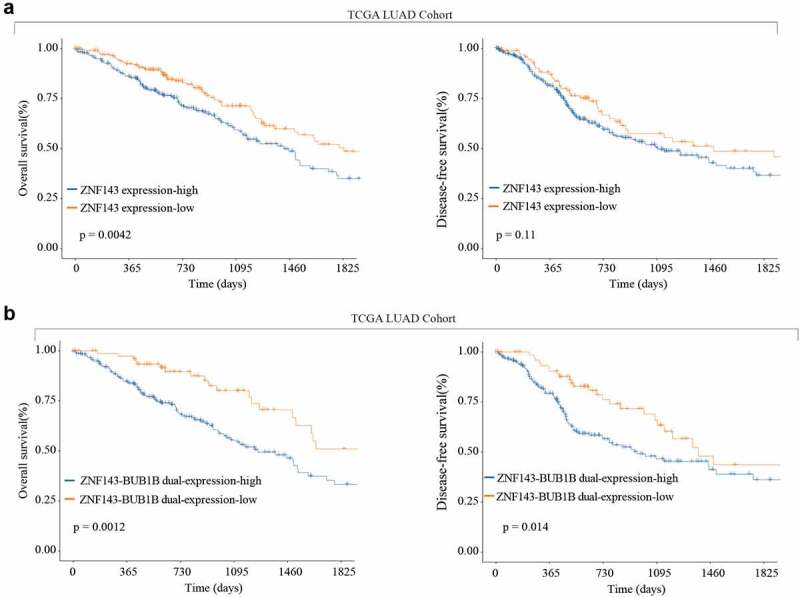
High ZNF143 expression indicates poor prognosis like BUB1B in LUAD. (a) Kaplan-Meier analysis of OS (left) and DFS (right) in LUAD patients with low or high ZNF143 expression. (c, d) Kaplan-Meier analysis of OS (c) and DFS (d) in LUAD patients with dual low or high ZNF143 and BUB1B expression.

Finally, we analyzed the TCGA LUAD cohort and found that high ZNF143 expression is associated with a shorter OS and DFS. Similarly, high expression of both ZNF143 and BUB1B revealed the same trend of a shorter OS and DFS as indicated by a higher expression of ZNF143 only (Figure 8). Therefore, these findings proved that ZNF143 and BUB1B play a co-oncogenes role in LUAD.

## Discussion

In the present study, we performed bioinformatic analysis with the TCGA and GEO databases and found that BUB1B is evidently overexpressed in LUAD tissues compared to paracancerous tissues. This was further confirmed at the protein level by IHC staining in 90 LUAD samples and their adjacent non-tumor tissues. Furthermore, the expression level of BUB1B was significantly correlated with the clinicopathological stages and survival outcomes. Modulating the expression of BUB1B led to change in various phenotypes of LUAD cells, including proliferation, migration, and invasion. Significantly, knocking-down BUB1B suppressed LUAD tumor growth in a xenograft model. Further mechanistic studies suggested that BUB1B promotes tumorigenesis by regulating glycogen metabolism and interacting with the transcription factor ZNF143. Therefore, our findings highlight a crucial oncogenic role of BUB1B and suggest its potential as a target for molecular therapy.

Glycogen metabolism plays an indispensable role in the development of cancers by intersecting with many other key metabolic pathways including glycolysis, Krebs cycle, lipid biosynthesis, and pentose shunt [21]. Thus, suppression of glycogen metabolism could provide a significant therapeutic advance in cancers [22]. Notably, multiple glycogen metabolism target genes are involved in LUAD progressions, such as GLUT1 [23] [24], LDHA [25], PKM2 [26], and HK2 [27]. Bioinformatic analysis showed that BUB1B expression is positively correlated with cell cycle and glycolysis pathway and inversely correlated with fatty acid metabolism. Indeed, we experimentally demonstrated that silencing BUB1B significantly reduces the expression of GLUT1, LDHA, PKM2, and HK2 in LUAD cell lines, implying that BUB1B might regulate LUAD progression through the glycogen metabolism signaling pathway. Further research is warranted to test the involvement of other key signaling pathways by which BUB1B functions in LUAD.

Prior work has documented that BUB1B is a kinase in the mitotic spindle checkpoint and has been implicated to be anomalously expressed in human cancers, such as ductal breast carcinoma [11], GBM [12], PDAC [13], and prostate cancer [15]. Xiong *et al* [28]. has also found that high expression of BUB1B along with CCNB1 and TTK can accelerate the progression of LUAD and lead to a shorter survival. However, mechanisms underlying BUB1B overexpression in LUAD are still not well understood. Through analyzing available datasets, we did not find any epigenetic or genetic modification in BUB1B of LUAD tissues, suggesting that BUB1B functions in a cell- or disease-context-dependent manner and that post-transcriptional regulation may be involved in BUB1B expression abnormality.

TFs regulate gene expression by binding to the promoter [29,30]. Herein, our bioinformatic analysis identified a TF, ZNF143, which binds to the promoter regions of *BUB1B* to regulate the expression of *BUB1B*. This result was in line with previous studies demonstrating that ZNF143 mediates the transcriptional activation of *BUB1B* in Drosophila SL2 cells [31]. Multiple lines of evidence has unveiled that ZNF143 regulates the progression of many tumors including gastric cancer [32], ovarian cancer [33], colon cancer [34], and hepatocellular carcinoma [35]. Silencing ZNF143 significantly resulted in a significant decrease in BUB1B mRNA and protein expression levels in LUAD cells, whereas overexpressing ZNF143 had the opposite effects. Moreover, the luciferase reporter assay demonstrated that ZNF143 markedly inhibited the relative luciferase activity in LUAD cells transfected with BUB1B-promoter WT, but not the mutated BUB1B-promoter. Additionally, Pearson’s correlation analysis revealed that there is a positive correlation between BUB1B and ZNF143 in LUAD. We further validated that ZNF143 knockdown had a similar effect on LUAD cancer cell proliferation, migration, and invasion as BUB1B knockdown, which was counteracted by BUB1B overexpression. Taken together, these results indicated that ZNF143 and BUB1B play a co-oncogenes role in the progression and tumorigenesis of LUAD.

In summary, our findings demonstrate that BUB1B is regulated by ZNF143 and that the ZNF143-BUB1B axis is likely to regulate the development and progression of tumor through glycogen metabolism signaling. Targeting such an axis may provide an effective approach against LUAD.

## Conclusion

BUB1B was upregulated in LUAD patients. LUAD patients with high BUB1B expression predicted poor outcome. Knocking-out BUB1B resulted in suppression of BUB1B LUAD malignancy in vitro and inhibition of tumor growth in vivo. Further analysis revealed that BUB1B regulates glycolysis in LUAD and interacting with ZNF143 in LUAD cells. The interaction was demonstrated by silencing ZNF143, which led to a decrease in proliferation, migration, and invasion in LUAD cells, whereas overexpressing BUB1B had the opposite effects. Our study suggested that the ZNF143/BUB1B axis plays a pivotal role in LUAD progression, which might be a potential target for LUAD management.

## Supplementary Material

Supplemental Material

## Data Availability

The datasets and supporting materials generated and/or analyzed during the current study are available from the corresponding author upon a reasonable request.

## Research highlights

- BUB1B is significantly up-regulated in LUAD tissues and predicts poor outcome in LUAD patients.
- BUB1B deletion suppresses LUAD malignancy in vitro and inhibits tumor growth in vivo.
- BUB1B regulates glycolysis in LUAD.
- ZNF143 modulates BUB1B in LUAD cells and ZNF143 enhances the tumorigenicity of LUAD cells by controlling BUB1B.
- High ZNF143 expression indicates poor prognosis like BUB1B in LUAD.
