# Supplementary material for: BUB1B (BUB1 Mitotic Checkpoint Serine/Threonine Kinase B) promotes lung adenocarcinoma by interacting with Zinc Finger Protein ZNF143 and regulating glycolysis
Source: Bioengineered. 2022 Jan 22;13(2):2471–85. doi: 10.1080/21655979.2021.2013108 (PMC8974056; doi:10.1080/21655979.2021.2013108)
Supplement: Supplemental Material [file KBIE_A_2013108_SM3984.zip › supplementary/Tab S1.docx]

**Table 1. Association between clinical features and BUB1B expression of LUAD patients.**

| **Variables Clinical features** | |  | | ***P-value*** |
| --- | --- | --- | --- | --- |
|  |  | **BUB1B High (n=51) BUB1B low (n=39)** | |  |
| Age (years) | ≤50 | 20 | 12 | 0.4068 |
|  | >50 | 31 | 27 |  |
| Gender | Male | 30 | 22 | 0.124 |
|  | Female | 21 | 7 |  |
| Smoking history | Smokers | 35 | 25 | 0.650 |
|  | Never smokers | 16 | 14 |  |
| TNM stage | Stage I/II | 26 | 36 | **0.02** |
|  | Stage II | 25 | 13 |  |
| Tumor size (cm) | ≤5 | 24 | 28 | **0.018** |
|  | >5 | 27 | 11 |  |
| Lymph metastasis | No | 22 | 27 | **0.003** |
|  | Yes | 29 | 12 |  |
